# The content and conduct of GP consultations for dermatology problems: a cross-sectional study

**DOI:** 10.3399/bjgp20X712577

**Published:** 2020-09-08

**Authors:** Emma Le Roux, Peter J Edwards, Emily Sanderson, Rebecca K Barnes, Matthew J Ridd

**Affiliations:** Centre for Academic Primary Care;; Centre for Academic Primary Care;; Bristol Randomised Trials Collaboration, Population Health Sciences, Bristol Medical School, University of Bristol, Bristol.; Centre for Academic Primary Care;; Centre for Academic Primary Care;

**Keywords:** decision making, shared, dermatology, primary care, self-management

## Abstract

**Background:**

Skin complaints are common in primary care, and poor outcomes in long-term conditions are often due to low adherence to treatment. Shared decision making and self-management support may help, yet there is little understanding of patient involvement or the support provided by GPs.

**Aim:**

To describe the content of primary care consultations for skin problems, including shared decision making practice, delivery of self-management advice, and follow-up.

**Design and setting:**

Cross-sectional study of video-recorded UK adult GP consultations and linked data.

**Method:**

A coding tool was developed and applied to all consultations with skin problems. Shared decision making was assessed using the observer OPTION^5^ scale.

**Results:**

A total of 45/318 consultations (14.2%) related to one or more skin problems, which were discussed alongside other problems in 71.1% (32/45) of consultations. Of the 100 different problems discussed in these consultations, 51 were dermatological. The mean amount of time spent on skin problems in the consultations was 4 minutes 16 seconds. Medication was recommended for 66.7% (34/51) of skin problems, with low shared decision making (mean OPTION^5^ score = 10.7). Self-management advice (verbal only) was given for 47.1% (24/51) of skin problems. Most skin problems (84.3%; 43/51) were not referred to secondary care; 32.6% (14/43) of the skin problems not referred were seen again in primary care within 12 weeks, of which 35.7% (5/14) follow-up appointments were not planned.

**Conclusion:**

In this study, skin problems were usually presented alongside other complaints and resulted in a medication recommendation. Shared decision making was uncommon and self-management advice not consistently given, with re-attendance for the same problem common. GPs’ training should reflect how frequently skin problems are seen and seek to improve patient involvement in decision making and support self-management.

## INTRODUCTION

Skin problems are the most common reason for a new presentation in primary care,^1^ and most patients with skin problems are managed exclusively in primary care.^2^ Historically, dermatology training for GPs has been limited,^2^^,^^3^ with GPs lacking confidence in diagnosing and managing skin conditions.^4^^,^^5^ Long-term skin problems can place a heavy burden on patients, including impaired health-related quality of life, reduced occupational productivity, and a high socioeconomic and psychological impact.^6^

Although most patients with the inflammatory skin problems of eczema, psoriasis, and acne have disease of mild to moderate severity, they require high levels of self-management.^7^^,^^8^ Effective topical treatments are the mainstay of care for most people, although treatment failure is common as a result of low adherence to treatments.^9^^,^^10^ There is a well-established evidence base for supported self-management in asthma where, like inflammatory skin conditions, treatment regimens change according to fluctuating symptoms.^11^ In comparison, there remains much uncertainty over the clinical and cost-effectiveness of interventions developed to date to improve self-management in long-term inflammatory skin conditions.^7^^,^^12^ A potential way to improve patient self-management might be through shared decision making around treatment decisions.^13^ Patients with long-term skin conditions desire shared management with healthcare professionals,^14^^–^^16^ and personalised care, with both verbal and written information.^14^^,^^16^^,^^17^

Although improved self-management and shared decision making have the potential to improve outcomes in long-term skin conditions, there has been little research exploring either the content or conduct of GP consultations with patients for skin problems. The aim of this study was to describe the content of routine primary care consultations for adults with skin problems, including the extent of observed shared decision making for treatment decisions, and the frequency and mode of delivery of self-management advice, followup arrangements, and record keeping.

## METHOD

### Data source

This was a cross-sectional study employing a content analysis of recordings held in the ‘One in a Million’ primary care consultations archive,^18^ which has previously been described in detail.^19^ In brief, 318 unselected adult consultations were available with permissions in place for analysis (comprising video-recordings, verbatim transcripts, electronic medical record entries, and GP and patient demographic and other survey data) with 23 GPs from 12 practices in the West of England, and that were collected between July 2014 and April 2015. ‘Unselected’ indicates consenting adults attending their GP surgery who could take part/have their consultation recorded and included in the archive. Consultations containing one or more skin problems were identified from existing coding of problems and issues discussed, undertaken in two previous studies.^19^^,^^20^

**Table:** How this fits in

In the UK, GPs diagnose and manage most skin problems, but treatment failure in long-term conditions is common as a result of low adherence with treatments. Although shared decision making for treatment decisions and supported self-management may improve disease outcomes and quality of life, little is known about how often this occurs in primary care consultations for skin problems. In this study, video-recordings of routine GP consultations were reviewed to explore these issues, and skin problems were found to present frequently alongside other complaints, and usually result in a medication recommendation. Shared decision making for treatment decisions was uncommon and self-management advice delivered by GPs not consistently given. Although most skin problems are not referred to specialist care, patients often reconsult for the same problem.

### Development of coding scheme

A novel coding scheme for content analysis was drafted by two authors as part of a masters dissertation,^21^ and refined iteratively by two authors using recognised methods of codebook development (see Supplementary Table S1 for details).^22^ Data were extracted on: duration of time spent on skin problems; co-occurrence with other conditions; observed medication recommendations; observed self-management advice; and observed followup arrangements and record keeping. Problem types were coded using the *International Classification of Primary Care*, version 2,^23^ through observation of GP–patient discussion (videos and transcripts). A problem was defined as the answer to the question, ‘What is wrong?’ from a published coding tool.^24^ Verbalised diagnosis was used ahead of presenting complaint where available.

Along with the novel coding scheme, the ‘observing patient involvement in decision making’ OPTION^5^ tool was also used to assess shared decision making.^25^ Shared decision making is a *‘... process in which clinicians and patients work together to clarify treatment, management or self-management support goals, sharing information about options and preferred outcomes with the aim of reaching mutual agreement on the best course of action’*.^26^ ^OPTION^^5^ (scale range 0–100) was chosen as the instrument to assess observed patient involvement in decision making because it has been used across a range of topics in primary care.^27^ Two coders read the OPTION^5^ manual and undertook online training with three encounter videos and quizzes before coding.^28^

There is no agreed definition of self-management, so for this study self-management was defined as *‘... day to day tasks an individual must undertake to control or reduce the impact of disease on physical health status’*^29^ and *‘... watching for changes, coping if symptoms worsen and knowing when to seek professional help’*.^13^ Similarly, follow-up arrangements may vary and therefore for this study are defined as a ‘verbalised discussion of the need to see another healthcare professional’.

### Measuring reliability

Two coders independently reviewed a random sample of 20% (9/45) of the consultations, and inter-rater reliability (IRR) statistics were computed for all codes using Stata (version 15.1). Percentage agreements and Cohen’s κ (weighted for partial agreements)^30^^,^^31^ were used to assess IRR for categorical data. For continuous data, quadratically weighted κ scores and two-way mixed-effects intraclass correlation (ICC) coefficient estimates for the absolute agreement between coders were calculated.^32^ Individual percentage agreement, κ, and ICC coefficients are reported in the supplementary material (see Supplementary Tables S2 and S3 for details). The mean agreement score for the novel coding tool (excluding duration of consultation and time on skin — reported as ICC coefficients) was 89% (κ = 0.74), demonstrating substantial IRR.^33^ ICC coefficients for all continuous data were excellent (range 0.94–1.00) using Munro’s classification,^34^ except for that of the total observer OPTION^5^ score, which was poor at 0.02. This was because of a lack of variation in the data (heavily skewed towards low/no shared decision making) and coders finding it hard to distinguish between OPTION scores of ‘no effort’ and ‘minimal effort’ of shared decision making. Across the 13 problems assessed during the IRR testing, when scores of no effort and minimal effort were combined, coders agreed that, for 62/65 (95%) of the individual OPTION item scores and for 10/13 (77%) of the total OPTION scores, there was no/minimal effort of shared decision making.

### Data extraction and analysis

The final version of the coding scheme was applied by one coder to the remaining 36 consultations identified as containing a skin problem. Data were collected into Microsoft Excel, which was then imported into Stata for descriptive statistical analysis, looking for associations between observed consultation and electronic medical record data, with descriptive statistics reported.

### Public and patient involvement

A mother of two children with eczema helped refine the aims of this study and reviewed the findings.

## RESULTS

### Characteristics of patients and consultations

A total of 45/318 (14.2%) consultations containing one or more skin problems were identified.^19^^,^^20^ The characteristics of the patients and their consultations are shown in Table 1. Two-thirds (30/45, 66.7%) of the patients were females and most (40/45, 88.9%) were of white ethnicity, with a range of participants by age and deprivation scores. A median (interquartile range) of 2 (1–3) problems were discussed per consultation, covering 100 different problems, of which 51 were skin-related. Mean duration of consultation time was 10 minutes:21 seconds (range 2:18 to 21:13), with a mean duration of 4 minutes:16 seconds (range 0:36 to 14:39) spent discussing the skin problem (41.2% of consultation time overall).

**Table 1.** Demographic and consultation characteristics (number of problems and duration of consultation) of study population, *n* = 45

**Patient characteristics**	**≥1 dermatological problems**	

	***n***	**%**
**Sex**		
Male	15	33.3
Female	30	66.7

**Patient age, years**		
18–34	16	35.6
35–54	9	20.0
55–74	13	28.9
≥75	6	13.3
Not reported	1	2.2

**Ethnic group**		
White	40	88.9
Other	3	6.7
Not reported	2	4.4

**IMD quintile**		
1^st^ (least deprived)	14	31.1
2^nd^	12	26.7
3^rd^	3	6.7
4^th^	4	8.9
5^th^ (most deprived)	12	26.7

**Consultation characteristics**		

**Number of problems per patient**		
1	13	28.9
2	16	35.6
3	11	24.4
≥4	5	11.1

**Median (IQR) number of problems**	2 (1–3)	

**Mean duration of consultation in minutes: seconds (range)**	10:21 (2:18–21:13)	

**Mean duration discussing skin problem in minutes: seconds (range)**	4:16 (0:36–14:39)	

IMD = Index Of Multiple Deprivation. IQR = interquartile range.

### Number and type of skin problems

Within the 45 consultations, 40/45 (88.9%) patients discussed one skin problem, 4/45 (8.9%) discussed two skin problems, and 1/45 (2.2%) discussed three skin problems. Dermatophytosis (6/51, 12%), atopic eczema (6/51, 12%), and lump/swelling localised (4/51, 8%) were most common (Table 2). In 71.1% (32/45) of consultations, skin problems were discussed alongside other problems, most frequently with musculoskeletal (8/100, 8%), cardiovascular (7/100, 7%), and psychological (6/100, 6%) complaints. In the 32 consultations where more than one problem was discussed, skin problems were most commonly raised as the second problem.

**Table 2.** Types of skin problems discussed in order of frequency

**ICPC-2 skin code**	**Frequency**	**%**
S74 Dermatophytosis	6	12.0
S87 Atopic eczema	6	12.0
S04 Lump/swelling localised	4	8.0
S03 Warts	3	6.0
S96 Acne	3	6.0
S99 Skin disease other	3	6.0
S16 Bruise/contusion	2	4.0
S29 Skin symptom/complaint other	2	4.0
S70 Herpes zoster	2	4.0
S82 Naevus/mole	2	4.0
S88 Dermatitis contact/allergic	2	4.0
S91 Psoriasis	2	4.0
S10 Boil/carbuncle	1	2.0
S12 Insect bite/sting	1	2.0
S21 Skin texture/symptom complaint	1	2.0
S23 Hair loss/baldness	1	2.0
S05 Lumps/swellings generalised	1	2.0
S06 Rash localised	1	2.0
S76 Skin infection other	1	2.0
S79 Neoplasm of skin benign/unspecified	1	2.0
S86 Dermatitis seborrhoeic	1	2.0
S09 Infected finger/toe	1	2.0
S90 Pityriasis rosea	1	2.0
S93 Sebaceous cyst	1	2.0
S97 Chronic ulcer skin	1	2.0
S84 Impetigo	1	2.0

**Total number of skin problems**	51	100

a % column may not sum to 100 because of rounding. ICPC = International classification of primary care.

### Diagnosis, medication recommendations, and observed shared decision making

GPs verbalised a diagnosis for 78.4% (40/51) of the skin problems, which was documented in the medical record in 85.0% (34/40) of cases.

Medication was recommended for 66.7% (34/51) of skin problems, with a mean of 1.5 medications (range 1–3). A total of 51 different medications were recommended, of which 56.9% (29/51) were new and 70.6% (36/51) were prescribed by the GP. Table 3 shows that the most common medication recommendations were emollients (15/51, 29.4%) and topical corticosteroid (TCS)/compound TCS-antimicrobials (14/51, 27.5%). Creams accounted for 40.0% (6/15) of emollient formulations recommended, and mild- and moderate-potency TCS accounted for 71.4% (10/14) of TCS/compound TCS-antimicrobial prescriptions.

**Table 3.** Number and type of medications observed to be recommended by GP for skin problem (in order of frequency)

**Code**		**Frequency**	**%^a^**
**Emollient/barrier preparation/soap substitute**		15	29.4
Cream		6	
Ointment		2	
Wash		2	
Gel		1	
Unclear		4	

**Topical corticosteroid (TCS)/compound TCS-antimicrobials**		14	27.5
*BNF* potency of TCS	Mild	5	
	Moderate	5	
	Potent	1	
	Very potent	1	
	Unclear	2	

Topical anti-infectives including antibacterial, antifungal, antiviral, and parasitical		5	9.8

Topical antiseptics		5	9.8

Oral antibiotic		4	7.8

Oral analgesia		3	5.9

Topical local anaesthetics and antipruritic		1	2.0

Topical preparations for acne/rosacea		1	2.0

Topical preparations for warts/calluses		1	2.0

Topical preparations for scalp conditions		1	2.0

Unclear		1	2.0

Total number of medications		51	100

a % column may not sum to 100 because of rounding. BNF = British National Formulary.

The mean OPTION^5^ total score for observer-rated shared decision making for skin problem treatment decisions was low at 10.7 (SD = 9.328; range 0–35).

### Observed self-management advice and follow-up arrangements

Self-management advice was observed to be offered in 47.1% (24/51) of all skin problems, 58.3% (14/24) of which were documented in the medical record. All self-management advice (24/24) was delivered verbally, with no written information or signposting to online resources.

Most skin problems (84.3%, 43/51) were not referred to secondary care, with ‘planned’ primary care follow-up verbalised in 37.2% (16/43) and ‘contingent’ followup in 30.2% (13/43) (Table 4). Of those skin problems not referred, ‘planned’ and ‘contingent’ primary care follow-up arrangements were documented in the medical record in 48.3% (14/29) of cases. A total 32.6% (14/43) of skin problems not referred to secondary care were seen again by the GP within 12 weeks of the index consultation, of which 35.7% (5/14) were unplanned [that is, there was no evidence of planned follow-up appointments] (Table 4).

**Table 4.** Re-consultation for the same skin problem within 12 weeks by primary care follow-up arrangements observed for skin problems NOT referred to secondary care, *n* = 43

**Re-consultation with GP for same skin problem within12 weeks**	**Primary care follow-up observed for skin problem, *n* (%)**			
	No evidence of planned follow-up	No planned follow-up but contingency approach	Follow-up with GP with time specified	Total
No	8 (32.0)	12 (48.0)	5 (20.0)	25
Yes	5 (35.7)	0 (<0.1)	9 (64.3)	14
No EMR data	1 (25.0)	1 (25.0)	2 (50.0)	4
**Total**	**14 (32.6)**	**13 (30.2)**	**16 (37.2)**	**43**

EMR = electronic medical record.

## DISCUSSION

### Summary

This study confirms that patients frequently present to their GP with skin problems. Most skin problems in the study were discussed alongside other complaints, with around one-third of the consultation time spent on them. A broad range of skin conditions were seen, with dermatophytosis, atopic eczema, and ‘localised swelling’ most common.

GPs often verbalised and documented a diagnosis for the skin problem. A medication was recommended for two-thirds of skin problems and more than half of these were new, with most being prescribed by the GP. Emollient and TCS/combined TCS–antimicrobials were most frequently advised, with GPs most often issuing mild or moderate strength of TCS. Although medication recommendations were common, shared decision making for treatment decisions was observed to be poor and self-management support delivered by GPs was low, with no written information or signposting to online resources offered.

Most skin problems were not referred to dermatology specialist care, with GPs offering planned or contingent followup arrangements in two-thirds of cases, which was documented for only half of the problems. Although most skin problems were not referred, re-attendance within 3 months of the index consultation was common.

### Strengths and limitations

To the authors’ knowledge, this is the first study to observe directly the content and conduct of consultations with GPs for skin problems. Use of video-recordings reduces problems associated with relying on self-reported behaviour and avoids an observer’s presence, which may potentially alter the consultation.^35^

Exploration of routine primary care was facilitated by the ‘One in a Million’ archive, where all patients seeing study GPs were invited to participate, thus providing good face validity. However, the sample is restricted in size (*n* = 45), with only Englishspeaking adults attending GP appointments in the West of England being eligible to participate. Despite there being a range of participants by age and deprivation, two-thirds of the sample were females, and 88.9% were of white ethnicity.

Subjective judgement was used to code problem types and record observed behaviours, although both coders are medically trained, which facilitated their understanding of the medical terminology, and the coding scheme was refined iteratively to ensure consistency of approach and understanding. However, the limitations of the reliability of the coding scheme are acknowledged. Overall, the results demonstrate that there was a low level of shared decision making when GPs were discussing skin problems, but the IRR testing demonstrated that it is not possible to comment precisely on whether GPs were demonstrating predominantly no shared decision making or minimal shared decision making, as specified by the OPTION^5^ tool.^25^

### Comparison with existing literature

The finding that, in the current study, 14.2% of GP consultations featured one or more skin problems is higher than a previously reported rate of 8.4% from a survey of general practices in the south east of Scotland,^36^ but lower than the 24% assessed through analysis of the Royal College of General Practitioners (RCGP) Weekly Returns Service consultation data from England and Wales in 2006.^2^ The mean duration of consultation time in this study of around 10 minutes is similar to previously reported national figures using data from 2014.^37^ However, it was not possible to quantify that only approximately one-third of total consultation time was spent discussing the skin problem. The median number of problems (two) discussed per consultation concurs with previous primary care research.^38^^,^^39^ The profile of skin conditions discussed in this study parallels that of previous primary care consultation analysis, where skin infections, eczema, and skin tumours (benign and malignant), were most common.^1^^,^^36^

The findings that emollients and TCS were most often recommended and issued as a prescription by GPs parallels national data on prescribing of medication for skin conditions.^2^ However, this prescribing pattern is likely to change since the introduction of prescribing restrictions for emollients in England in 2017.^40^

Although healthcare policy and dermatology clinical guidelines advocate shared decision making for treatment decisions,^41^^–^^43^ low levels of patient involvement were observed (mean OPTION^5^ score of 10.7). Previous research in the US and Germany using OPTION^5^ has also reported low levels of observed shared decision making in primary care consultations, with mean total scores of 27.2^27^ and 11.8.^44^ In contrast, studies in secondary care have observed higher shared decision making (mean total OPTION^5^ score range = 31.0 to 60.6).^45^^–^^47^ Lower scores in primary care could be because of better doctor–patient continuity, where observation of serial consultations may be required to capture ‘disseminated’ shared decision making.

Despite current dermatology guidelines recommending the use of personalised self-management plans and signposting to high-quality information,^42^^,^^43^^,^^48^ self-management advice was observed not to be consistently offered by GPs to patients, with no written information offered. Although delivery of self-management advice may be less applicable for some of the problems observed in this study, for example, a self-limiting rash, it is common for patients to have poor recall for medical information,^49^ which can be improved by delivery of supportive written information.^50^ Previous research has shown that while self-management support is valued by GPs, it is not prioritised in time-limited consultations for fear of disrupting the clinician–patient relationship, and clinicians may be more inclined to focus on biomedical aspects of care.^51^^–^^53^ Other barriers identified to GPs facilitating self-management include lacking confidence to share control, ability to provide ongoing support, and concerns that patients may use medications inappropriately and not seek help when needed.^51^^,^^54^

The secondary care referral rate from this study of 15.7% is comparable with a cross-sectional study of Scottish primary care consultations for skin problems, where 14% of patients were referred for specialist assessment.^36^ This is higher than that previously reported from national estimates in England and Wales of 6.1% in 2006/2007.^2^ The RCGP Weekly Returns Service data reported approximately two consultations per episode of skin disease in primary care,^2^ and frequent re-attendance (defined as three or more consultations for the skin problem) was noted in 25% (range 14–45%) of Scottish primary care consultations for skin problems.^36^ In comparison, the current study found that one-third of the skin problems not referred to secondary care were seen again within 3 months, one-third of which were unplanned appointments. Reasons for re-attendance with skin problems have not been well explored, although the Scottish primary care consultations for skin problems study identified recurring treatment issues, which included a reluctance to prescribe appropriate potencies of TCS in eczema.^36^

### Implications for research and practice

Research is required to answer whether there are common recurring issues for re-attendance with skin problems in primary care, which may inform dermatology education and interventions to support shared decision making and self-management support. Further work is needed on the development of interventions aimed at increasing shared decision making and self-management support in primary care consultations for skin problems, and their evaluation should be longitudinal to capture decision making over a series of consultations. Use of validated shared decision making instruments alongside patient outcomes would enable a clearer understanding of whether shared decision making is leading to an improvement in outcomes.

Meanwhile, this study highlights the importance of dermatology in primary care and the challenge to shared decision making and delivery of self-management support, where skin problems are frequently presented alongside other conditions in time-limited consultations. However, the low levels of observed shared decision making around treatment decisions and self-management support suggests that a cultural change is needed among practitioners, moving away from solely biomedical approaches to personalised care planning. Clinicians’ training^55^ and assessments should include both psychosocial and physical aspects, and address patient preferences for treatment decisions. An example of training available to practitioners to support self-management in patients with psoriasis is the ‘Pso Well’ training course.^56^

Preparation of personalised self-management plans such as one developed by the authors in 2018 for eczema in children,^57^ and referral to high-quality supportive information alongside review appointments, potentially with other primary healthcare professionals including nurses and pharmacists, could facilitate better self-efficacy and health outcomes.
